# Falcarindiol Purified From Carrots Leads to Elevated Levels of Lipid Droplets and Upregulation of Peroxisome Proliferator-Activated Receptor-γ Gene Expression in Cellular Models

**DOI:** 10.3389/fphar.2020.565524

**Published:** 2020-08-28

**Authors:** Camilla Bertel Andersen, Anders Runge Walther, Emma Pipó-Ollé, Martine K. Notabi, Sebastian Juul, Mathias Hessellund Eriksen, Adam Leslie Lovatt, Richard Cowie, Jes Linnet, Morten Kobaek-Larsen, Rime El-Houri, Morten Østergaard Andersen, Martin Aage Barsøe Hedegaard, Lars Porskjær Christensen, Eva Christensen Arnspang

**Affiliations:** ^1^Department of Chemical Engineering, Biotechnology and Environmental Technology, University of Southern Denmark, Odense, Denmark; ^2^The Maersk Mc-Kinney Moller Institute, University of Southern Denmark, Odense, Denmark; ^3^Mads Clausen Institute, University of Southern Denmark, Odense, Denmark; ^4^Department of Clinical Research, University of Southern Denmark, Odense, Denmark; ^5^Department of Surgery, Odense University Hospital, Odense, Denmark; ^6^Department of Chemistry and Bioscience, Faculty of Engineering and Science, Aalborg University, Esbjerg, Denmark

**Keywords:** falcarindiol, lipid droplets, cholesteryl esters, PPARγ, ABCA1, colorectal cancer, type 2 diabetes, Raman spectroscopy

## Abstract

Falcarindiol (FaDOH) is a cytotoxic and anti-inflammatory polyacetylenic oxylipin found in food plants of the carrot family (Apiaceae). FaDOH has been shown to activate PPARγ and to increase the expression of the cholesterol transporter ABCA1 in cells, both of which play an important role in lipid metabolism. Thus, a common mechanism of action of the anticancer and antidiabetic properties of FaDOH may be due to a possible effect on lipid metabolism. In this study, the effect of sub-toxic concentration (5 μM) of FaDOH inside human mesenchymal stem cells (hMSCs) was studied using white light microscopy and Raman imaging. Our results show that FaDOH increases lipid content in the hMSCs cells as well as the number of lipid droplets (LDs) and that this can be explained by increased expression of PPARγ2 as shown in human colon adenocarcinoma cells. Activation of PPARγ can lead to increased expression of ABCA1. We demonstrate that ABCA1 is upregulated in colorectal neoplastic rat tissue, which indicates a possible role of this transporter in the redistribution of lipids and increased formation of LDs in cancer cells that may lead to endoplasmic reticulum stress and cancer cell death.

## Introduction

Cancer and diabetes constitute a heavy burden to societies and health care systems globally. Therefore, it is of great importance to develop new ways of preventing and treating these diseases. Type 2 diabetes is furthermore associated with increased risk of some types of cancer, such as colorectal cancer (CRC) clearly indicating the existence of biologic links between these diseases (Yang et al., 2005; Giovannucci et al., 2010; Lecarpentier et al., 2017). We have recently demonstrated that the dietary bioactive polyacetylenic oxylipins falcarinol (FaOH) and falcarindiol (FaDOH) have a dose dependent chemopreventive effect on colorectal neoplastic lesions in a rat model of CRC (Kobaek-Larsen et al., 2017; Kobaek-Larsen et al., 2019). We have furthermore shown that this antineoplastic effect is most likely due to inhibition of pro-inflammatory markers in the NF-κB signaling pathway such as TNFα, interleukin 6, and cyclooxygenase-2 (Kobaek-Larsen et al., 2019). These polyacetylenes have furthermore shown to stimulate basal and insulin-dependent glucose-uptake in cell cultures (El-Houri et al., 2015a). Therefore, these bioactive secondary metabolites seem to have a preventive effect on the development of both cancer and type 2 diabetes. The question is whether this can be explained by a common mechanism of action and, if so, how this can be utilized to develop dietary supplements and/or drugs to prevent the development of these diseases.

FaOH and FaDOH are found in several important dietary vegetables from the Apiaceae family including carrots, where they play an important role as defence compounds against fungal infections (Christensen and Brandt, 2006). Several meta-analysis studies on carrot consumption have indicated that carrots play a central role as a protecting vegetable, against development of diﬀerent types of cancers (Xu et al., 2014; Fallahzadeh et al., 2015; Chen et al., 2018). A recent prospective cohort study, examining the risk of being diagnosed with CRC as predicted by intake of carrots in a Danish population of 57,053 individuals with a long follow-up confirmed the cancer preventive effect of this vegetable. This preventive effect of carrot intake on CRC development is possibly due to the content of FaOH and FaDOH (Deding et al., 2020).

The cytotoxicity and anti-inflammatory effect of FaOH and FaDOH has been demonstrated *in vitro* in numerous investigations. From these investigations, it appears that FaOH is more cytotoxic than FaDOH, whereas FaDOH show greater anti-inflammatory effect than FaOH (Christensen and Brandt, 2006; Purup et al., 2009; Um et al., 2010; Christensen, 2011; Kobaek-Larsen et al., 2019). FaOH and FaDOH are highly alkylating compounds due to their unsaturated electrophilic system being able to bind covalently to proteins and other biomolecules and this may to some extent explain their cytotoxic and anti-inflammatory activity (Christensen, 2020). It has for example been demonstrated that these polyacetylenes are able to inhibit the efflux protein ABCG2 involved in breast cancer chemotherapy resistance (Tan et al., 2014) and mitochondrial aldehyde dehydrogenase (ALDH2) (Heydenreuter et al., 2015) by covalent binding to these proteins. A reduction in the activity of ALDH2 may lead to oxidative stress and endoplasmic reticulum (ER) stress causing cell cycle arrest and apoptosis (Liao et al., 2012; Iurlaro and Muñoz-Pinedo, 2016). The electrophilic nature of FaOH and FaDOH also enables them to activate the Keap1-Nrf2-signaling pathway (Ohnuma et al., 2009; Ohnuma et al., 2010; Stefanson and Bakovic, 2018). The Keap1-Nrf2 pathway regulates the expression and formation of a battery of antioxidant, anti-inflammatory, and cytoprotective phase 2 enzymes and therefore this pathway may also contribute to the understanding of the chemopreventive effects of these polyacetylenes.

Furthermore, FaDOH has been shown to act as partial agonist of the adipogenic transcription factor PPARγ (Atanasov et al., 2013; El-Houri et al., 2015a; El-Houri et al., 2015b). PPARγ is known to increase glucose uptake and lipolysis of adipocytes, and explains why FaDOH has been found to induce basal glucose uptake and insulin-stimulated glucose uptake in adipocytes and myotubes as well as lipolysis in adipocytes (El-Houri et al., 2015a). In addition, PPARγ has also shown to play an important role in the regulation of cancer cell growth due to its anti-proliferative and pro-apoptotic properties (Tachibana et al., 2008). PPARγ agonists are for example known to inhibit the development of CRC (Sarraf et al., 1998), and therefore FaDOH may also exert its chemopreventive effects through activation of PPARγ. Activation of PPARγ can also lead to increased expression of the ATP-binding cassette transporter A1/cholesterol exporter (ABCA1) (Chawla et al., 2001) and it has been shown that FaDOH promote ABCA1 gene expression and increases the stability of ABCA1 in macrophages by inhibition of proteolysis leading to enhanced cholesterol efflux (Wang et al., 2017). The ABCA1 protein mediates the transfer of cellular cholesterol across the plasma membrane to high-density lipoprotein (HDL) and ABCA1 is thus involved in cholesterol efflux through HDL formation (Wang and Tall Alan, 2003). Cancer cells are fast proliferating cells that require high levels of cholesterol for membrane biogenesis and other functional needs and increased cholesterol biosynthesis is therefore a hallmark of many cancers. In particular, the increase of intracellular cholesterol levels appears to inhibit cell death of cancer cells facilitating cancer cell survival (Huang et al., 2020). The anticancer function of ABCA1 in human cancer cells has been demonstrated and is probably related to its ability to decrease the content of mitochondrial cholesterol resulting in the release of cell death-promoting molecules (Smith and Land, 2012). This is also consistent with the fact that tumors show increased levels of cholesterol compared to normal tissue and therefore cholesterol lowering in cancer cells has been suggested as a potential anticancer strategy (Smith and Land, 2012; Gu et al., 2019).

Accumulation of excess cholesterol in the form of cholesteryl esters is another characteristic of cancer cells. The cholesteryl esters are usually stored in lipid droplets (LD) serving as a readily available reservoir for neutral lipids (Tirinato et al., 2017; Cruz et al., 2020). Although an accumulation of lipid droplets in cancer cells can lead to increased proliferation and aggressiveness of tumors (Gu et al., 2019; Cruz et al., 2020), it appears to depend on the total amount of cholesterol accumulated in cancer cells. It has for example been shown that some polyunsaturated fatty acids, such as docosahexaenoic acid, cause a redistribution of cholesterol from intracellular compartments (mitochondria and ER) to polyunsaturated-enriched lipid droplets but not in an increase of total cholesterol (Jakobsen et al., 2008). This change in cholesterol metabolism causes functional depletion of cholesterol in these intracellular compartments leading to ER stress, and thus to the induction of cell cycle arrest and/or apoptosis of cancer cells (Jakobsen et al., 2008; Huang et al., 2020). The mechanism by which FaDOH promotes cancer cell death has been shown to be due to excessive ER stress in colon cancer cells but not in normal colon epithelial cells (Jin et al., 2012). FaDOH has also been shown to induce ER stress in breast cancer cells (Lu et al., 2017). Thus, it appears that FaDOH may have multiple targets for its chemopreventive effects. Some of these targets could be related to changes in the biosynthesis, metabolism, and redistribution of cholesterol in cancer cells due to for example increased expression of ABCA1 and formation of LDs leading to ER stress and cell death.

Increased cholesterol levels are also associated with type 2 diabetes and it has been demonstrated that type 2 diabetes is associated with reduced ABCA1 gene expression (Patel et al., 2011). Thus, regulation of lipid and cholesterol formation by FaDOH through PPARγ activation could be a common mechanism of action of this polyacetylene that can contribute to both its anticancer and antidiabetic effect. This encouraged us to investigate how FaDOH may exert its function on cellular level with regard to lipid and cholesterol formation and accumulation in cells, in order to obtain more insight into its possible mechanisms of action. This information could be used to develop anticancer and antidiabetic drugs using FaDOH as a lead compound as well as to identify dietary sources for the prevention of cancer and type 2 diabetes.

In this study, we used two human cell lines; human mesenchymal stem cells (hMSCs) and human colon adenocarcinoma cells (HT-29) to investigate the effect of FaDOH on lipid metabolism at the cellular level. hMSCs are relevant cells as they play a key role in obesity (Matsushita and Dzau, 2017) and are able to generate new adipose tissue through adipogenic differentiation driven by PPARγ (Cristancho and Lazar, 2011). hMSCs have also shown a potential role in the treatment of diabetes (Moshtagh et al., 2013) and have shown to be involved in the progression of cancers (Lopatina et al., 2016; Ridge et al., 2017). In this study, we have investigated the effect of FaDOH on hMSCs using Raman spectroscopy, white light microscopy to get information of the faith of FaDOH in cells and its effect on lipid formation. Furthermore, we utilized RT-qPCR to study the effect of FaDOH on gene expression in HT-29 cells as well as FaDOH/FaOH (ratio 1:1) on the expression of ABCA1 in neoplastic rat tissue, from the recent rat studies (Kobaek-Larsen et al., 2017; Kobaek-Larsen et al., 2019). We found that treatment with FaDOH leads to increased lipid content and the number of lipid droplet (LD) in the cells, moreover FaDOH treatment increased expression of PPARγ2 in HT-29 cells and an increased ABCA1 expression was observed in tumor rat tissue.

## Materials and Methods

### Preparation of Falcarindiol

FaOH and FaDOH was isolated from carrots (*Daucus carota* ssp*. sativus* cv. Miami) by column chromatography and preparative HPLC and identified by spectroscopic and spectrometric methods as described previously (Kobaek-Larsen et al., 2017) with a purity >98%. FaDOH was stored in EtOH at -20°C in 250 mM until administrated to cells.

### Cell Cultures

The human colon adenocarcinoma (HT-29) cell line was provided by instituto Cantonale di Patalogia (ICP), Locarno, Switzerland. HT-29 cells and the human mesenchymal stem (hMSC) cell line (Simonsen et al., 2002) were grown in McCoy’s 5A modified with L-glutamine and sodium bicarbonate (Sigma-Aldrich) and complete MEM medium (Gibco, Copenhagen, Denmark), respectively, both with 10% fetal bovine serum (FBS, Sigma, Copenhagen, Denmark) and 1% penicillin/streptomycin (Amresco, Herlev, Denmark). The cells were grown in a humidified chamber at 37°C with 5% CO_2_.

### Viability Test

Cells were seeded at a concentration of 5,000 cells/well in 96 well plates in 100 µl of medium. After 24 h, the medium was replaced with fresh medium that included FaDOH at different concentrations. After a further 72 h the cells were visualized using an inverted microscope (Motic AE31) with a 10× objective and were then photographed. The wells were then assessed for viability using a resazurin viability assay (Tox8, Sigma Aldrich) according to the manufacturer’s instructions. Resazurin solution was mixed with fresh medium at 1:9 and 100 µl of this mixture was added to each well. After 3 h of incubation 80 µl was transferred to a new 96 well plate and absorbance at 600 nm and 690 nm was read using a 96 well plate reader (BioTek Epoch). Background absorbance at 690 nm was subtracted from absorbance at 600 nm to get dye specific absorbance. Viable cells convert the dye into a red product and 100% viability was set to the difference in absorbance between non-treated cells and unreacted dye that had been incubated in wells without cells. Percent viability was then calculated for the samples as the fraction of color change compared to the non-treated cells.

### Preparation of Samples for Imaging and Microscopy

Borosilicate glass coverslips (VWR) were sterilized in 98% ethanol and air dried under sterile conditions and placed in 6 well plates. 50,000 cells (hMSC Tert4, p44) were seeded on the 18 mm square or 25 mm round coverslips in 3 ml complete MEM medium (Gibco, Copenhagen, Denmark) with 10% fetal bovine serum (FBS, Sigma, Copenhagen, Denmark) and 1% penicillin/streptomycin (Amresco, Herlev, Denmark) and grown in a humidified chamber at 37°C with 5% CO_2_. After 24 h, the medium was removed and replaced with complete MEM medium (control) or complete MEM medium with 5 μM FaDOH. After 1 h, 5 h, and 24 h the wells were rinsed twice with PBS and cells were fixed in 4% formaldehyde for 10 min at room temperature. The coverslips were then stored in PBS and in darkness at 4°C until use. For each time point, three biological replicates were prepared and fixed for both controls cells and cells exposed to FaDOH.

### Raman Imaging

An in-house build Raman imaging setup was utilized for cell imaging. The Raman excitation source comprises a 532 nm laser (Laser Quantum, Ventus 532 nm, Stockport Cheshire) coupled *via* free space optics into an Olympus BX60 microscope (Hamburg, Germany) focusing the laser onto the sample through a 100X, NA=1 water immersion objective (Olympus) resulting in an effective spot diameter around 650 nm. The backscattered Raman signal was collected by the microscope and directed *via* a 105 μm fiber (Thor Labs, GmbH, Germany) to an Acton SpectraPro 2500i f/6.5, 600 l/mm spectrograph with a 100 μm slit and a Princeton Instruments PIXIS 400F 1340×400 Pixel CCD camera (Trenton, NJ) operated at −75°C resulting in a 3−6 cm^-1^ spectral resolution. Cells were mapped utilizing a motorized stage at 1 μm steps (best spatial resolution of stage), and spectra were collected with an excitation power of 100 mW (using water immersed samples), 1 accumulation and 1 s acquisition time per spectrum. No sample degradation was noticed using this power density. For each control and FaDOH a minimum of three cells were imaged for each time point and biological replicate resulting in at least 9 cell maps per time point.

All spectral processing was performed in MATLAB (The Mathworks, Inc., Natick, MA, US) and follows the methods described in the literature (Hedegaard et al., 2016). For each measured cell a data cube was obtained comprising a number of spectra each corresponding to a spatial position in the cell image. Preprocessing included removal of cosmic rays and the specific solvent and substrate background that are unrelated to the biochemical information. The background correction was performed on each Raman image using EMSC-SIS with reference spectra extracted from the individual dataset by k-means clustering. Spectra were offset corrected by subtracting the average Raman intensity in the wavenumber range 2,429 cm^-1^ to 2,513 cm^-1^ from the entire spectrum. Initially the spectral unmixing algorithm N-FINDR was applied to each individual image using 1 to 6 endmembers. The number of endmembers were chosen to maximize the number of biochemically meaningful spectra through peak assignment. False color images of the cells were constructed by identifying the biochemical content from the “pure” endmember spectra and assigning them with a specific color. For direct comparison of biochemical content in the cells all images were unfolded into a single matrix and all spectra were normalized using EMSC. N-FINDR analysis was then conducted on the entire matrix focusing only on the spectral region of the CH-stretch (2760 cm^-1^ to 2983 cm^-1^) using 6 endmembers. All spectra were smoothed using a 10-point average smoothing filter prior to the analysis. Relative area quantification for specific chemical components were then conducted using an abundance value threshold of 0.2.

### White Light Microscopy

An Olympus IX81 microscope was used to acquire images with the 150× (NA 1.45) oil objective and the DIC (Differential Interference Contrast) channel. Fixed untreated control cells and 24 h FaDOH treated cells were imaged. Fifty images were analyzed for both sets of samples; LDs were counted with ImageJ in every cell. A non-parametric Wilcoxon Rank Sum test was executed to compare the two sets of samples, which did not follow a normal distribution. *P*-values < 0.05 were considered significant.

### PPARγ Gene Expression in HT-29 Cells

The expression level of the gene PPARγ2 in HT-29 cells was investigated with Real-time quantitative PCR (RT-qPCR). 300.000 HT-29 cells/well were seeded in 6-well plates the day before FaDOH treatment. Then media was removed and 5 µM FaDOH in 3 ml media was added to the cells and the cells were incubated for additional time. Each well in the 6-well plate, had a different FaDOH treatment time. The following times were used: 0 min (control), 10 min, 1 h and 24 h. Control (0 min) experiment was performed with media without FaDOH added. The 24 h treatment was starting 24 h before 0 min treatment and so on. Thereby all the treatments in the same plate were stopped at the same time. The treatment was stopped by harvest of the cells using 500 µl of QIAzol Lysis Reagent (Qiagen) and shaking at 900 rpm for 2 min. The RNA was extracted using EconoSpin column purification and subsequently 0.3 µg RNA was converted into complementary DNA (cDNA). The synthesized cDNA was then analyzed with RT-qPCR using GoTaq^®^ Probe qPCR master mix and MyGo Mini instrument. Housekeeping gene was ribosomal protein large P0 (RPLP0). The primers (Merk) and fluorescence probes (PentaBase, Odense) can be found in **Table 1**.

**Table 1:** DNA sequence of primers and probes for RT-qPCR of HT-29 cells and for neoplastic tissue from rat.

Gene	Forward Primer (5’-3’)	Revers Primer (5’-3’)	Probe (5’-3’)
PPARγ2 (human)	AGCAAACCCCTATTCCATGCT	ATCAGTGAAGGAATCGCTTTCTG	TATGGGTGAAACTCTGGGAGA
RPLP0 (human)	GGCGACCTGGAAGTCCAACT	CCATCAGCACCACAGCCTTC	ATCTGCTGCATCTGCTTGGAGCCCA
ABCA1 (rat)	TGCCCCACCATGTAAAGTAC	CCCCAGACATAGCGCATATC	CAAGGZATGZGGTZACTGGGZACC
GUSB (rat)	CGTACCAGCCACTATCCCTA	AGACACGTTGCCAAAACTCT	TGGTCATCGZATGZAGTGTCCC

Up- or downregulation of the genes was quantified by means of fold changes using the comparative CT method (eq. 1) (Livak and Schmittgen, 2001; Schmittgen and Livak, 2008).

(eq. 1) $$2^{-\Delta \Delta CT}=2^{-[(CT,GOI-CT,HK)treated\;sample-(CT,GOI-CT,HK)control\;sample]}$$

where, CT is the threshold cycle, GOI is the gene of interest and HK is the housekeeping gene. The equation gives a gene expression normalized to the housekeeping gene and compared to untreated control. If the treated sample ΔCT is greater than the control ΔCT, the value of 2^*−ΔΔCT*^ will be less than 1 implying a decrease in the gene expression, hence a value higher than 1 means an increase in gene expression. qPCR was performed in duplicates for each sample and the experiment was repeated three times (n=3). Data are presented as mean ± standard deviation (SD). Data were analyzed using Student *t*-test (two-tailed) and * marks *P* < 0.05.

### Gene Expression in Neoplastic Rat Tissue

Animal studies were approved by the Central Animal Experimentation Inspectorate in Denmark (License no. 2015-15-0201-00708). Five weeks old male rats (F344 strain), with a certified health report, were purchased from Charles River Laboratories Copenhagen A/S (Lille Skensved, Ejby, Denmark). The rats were acclimatized for one-week where after they were divided into two groups. Group 1 was fed standard rat diet (SRD) (Altromin 1321, Brogaarden, Lynge, Denmark) and group 2 was fed SRD supplemented with FaOH and FaDOH as previously described (Kobaek-Larsen et al., 2019). Rats were housed as described in earlier studies (Kobaek-Larsen et al., 2017).

The gene expression of ABCA1 in rat tissue from colon biopsies was analyzed using RT-qPCR. The biopsies include neoplastic tissue from the group 1, receiving SRD and size-matched neoplastic tissue from the group 2 receiving rat diet supplemented with 7 µg FaOH g^-1^ feed and 7 µg FaDOH g^-1^ feed. RNA from the tissue was extracted using QIAzol, purified using EconoSpin columns, converted into cDNA, and finally evaluated using RT-qPCR. Multiplex RT-qPCR was performed with the housekeeping gene beta-glucuronidase (GUSB) as an internal reference. ABCA1 was measured in four replicates, run pairwise. Up- or downregulation of the ABCA1 was quantified using the comparative CT method as described under gene expression in HT-29 cells. Primers (MERK) and probes (PentaBase, Odense, Denmark) can be found in **Table 1**. Statistical analysis was performed using SAS JMP. Pro 13.0.0. Data are presented as mean ± standard deviation (SD). Data were analyzed using Student *t*-test (two-tailed) and *** marks *P* < 0.001.

## Results

### FaDOH Is Cytotoxic

**Figure 1:**
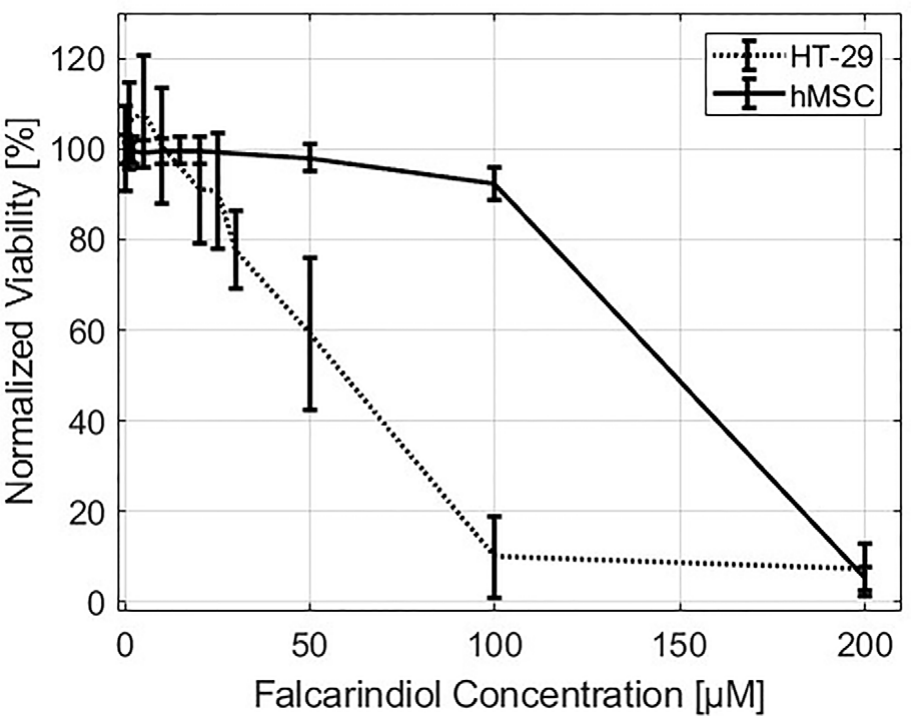
Viability. Normalized viability of HT-29 (dotted line) and human mesenchymal stem cells (hMSCs) (solid line) as function of falcarindiol (FaDOH) concentration in the cell culture medium measured after three days of exposure.

We performed a viability test on hMSCs and HT-29 grown for 72 h in cell culture medium containing different concentrations of FaDOH. Close to 100% hMSC viability was observed for concentrations between 1 to 20 µM FaDOH and only concentrations above 50 µM exhibited a toxic effect on the cells (**Figure 1**). For HT-29 cells, concentrations above 20 µM FaDOH decreased the viability. 5 µM FaDOH was subsequently used for the experiments in order to treat the cells with sub-toxic concentrations.

### FaDOH Changes the Lipid Content of the Cell

**Figure 2:**
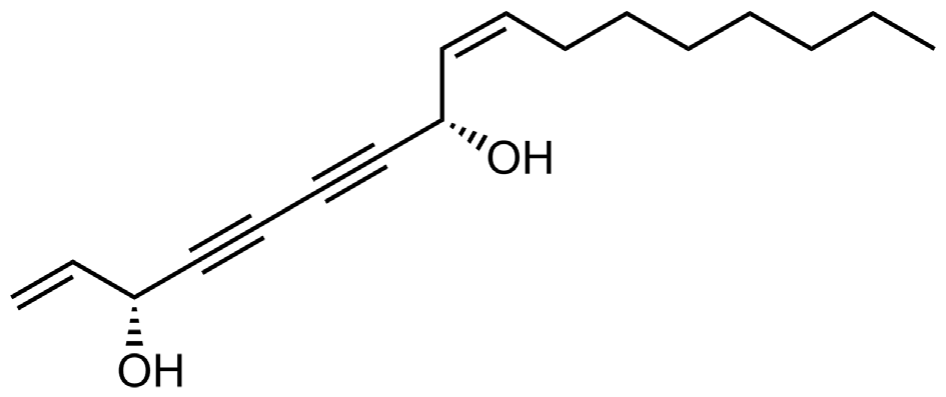
Chemical structure of (3*R*,8*S*)-falcarindiol (FaDOH) isolated from carrots.

To study the chemical variations within the cells Raman spectroscopy was used. Raman spectroscopy is a noninvasive, powerful label-free and chemical-specific technique that has been shown to be able to detect changes in lipid metabolism in cells with high specificity and reliability, and is widely used for the diagnosis of various cancers by analyzing the abundance of lipids such as polyunsaturated fatty acids in cancer cells (You et al., 2016). Raman spectroscopy mapping is an optical method that provides combined chemical and spatial information and thus allows label free investigation of chemical changes in sub-cellular locations (Krafft et al., 2009). Raman spectroscopic mapping is therefore a powerful label-free technique for studying intracellular trafficking of FaDOH as well as the effects of FaDOH on the same cells. FaDOH contains multiple carbon-carbon triple bonds (**Figure 2**) and is therefore potentially traceable with Raman spectroscopy as these possess unique Raman signatures (Baranska et al., 2005). In this study, however, we did not observe signal from FaDOH in the cell samples, which could be due to concentrations below detection limit. Although, we were not able to show that FaDOH is entering the cells in the present study, it is known from pharmacokinetic studies both in rats and in humans after oral administration of FaDOH that this polyacetylene is rapidly absorbed, which indicates that FaDOH is able to cross cell membranes (Christensen, 2020). Thus, this substantiate our hypothesis that FaDOH is able to exert a biological activity in cells *in vitro* as well as *in vivo*.

**Figure 3:**
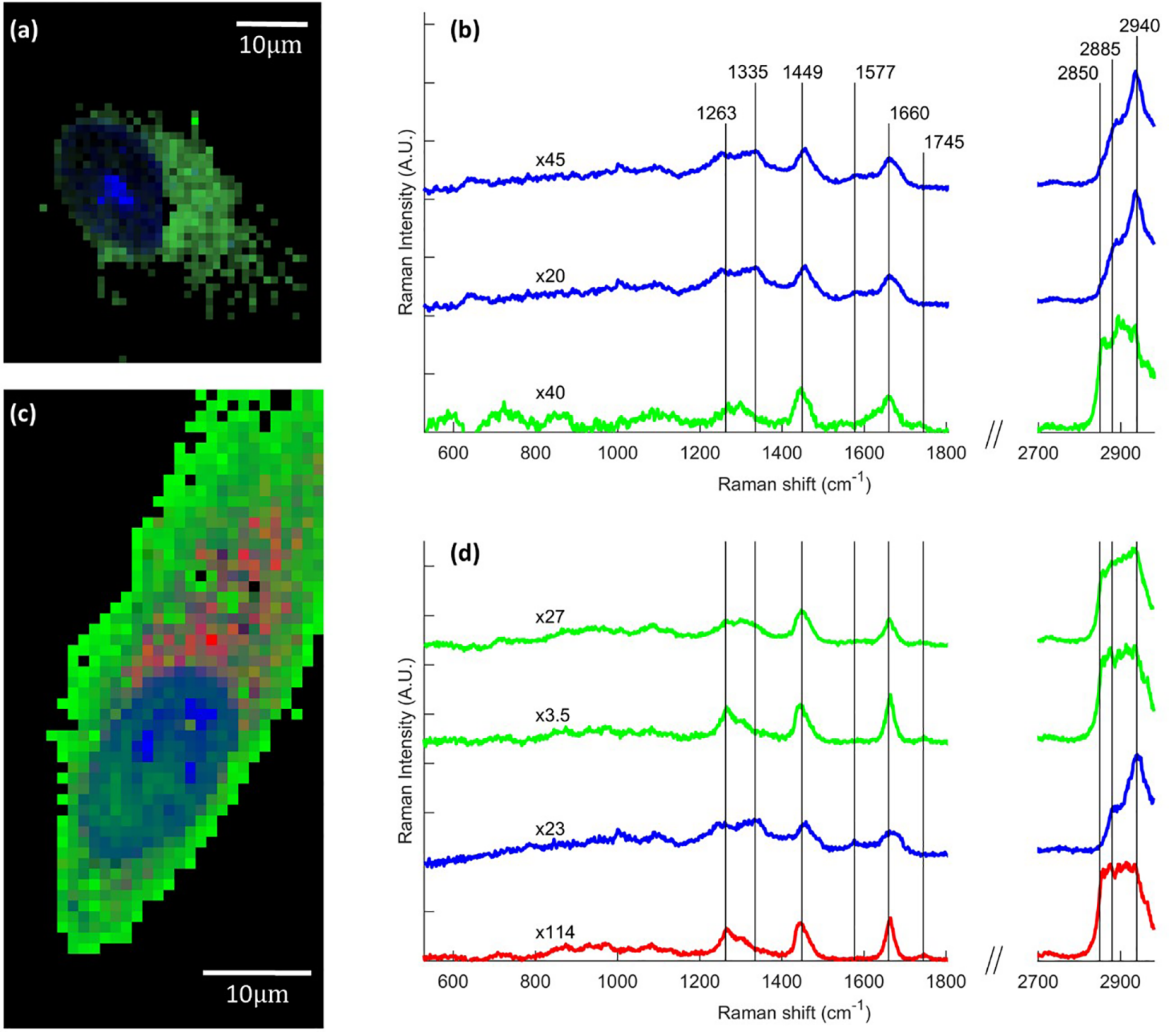
Raman images of human mesenchymal stem cells (hMSCs), showing chemical variation due to falcarindiol (FaDOH) treatment. Representative examples of false color images and corresponding endmember Raman spectra for control cell **(A, B)** and 24 h FaDOH treated cell **(C, D)** produced using the spectral unmixing algorithm N-FINDR on the individual cell dataset. Endmember spectra are normalized (scale values indicated by x#) and truncated in the silent region (1,800–2,700 cm^-1^) for clarity. Each pixel in the images is represented by a combination of colours depending on the abundance value of an endmember. Blue, green and red endmembers contain bands associated with nucleus, cytoplasm and lipids respectively. Scale bars are 10 µm.

Raman imaging was performed on cells grown on borosilicate coverslip and treated with 5 µM FaDOH for 0, 1, 5 and 24 h before the cells were fixed and imaged. Spectral unmixing N-FINDR (Hedegaard et al., 2011) analysis of the Raman images was applied, producing pseudo pure spectra (endmembers) from the dataset describing the biochemical composition of the cell (Kallepitis et al., 2017). This was done on all individual Raman maps producing false colour images in which each pixel is represented by a combination of colours depending on the abundance value of an endmember spectrum. Characteristic endmember spectra containing vibrational bands representing nucleus (blue) and cytoplasmic content (green) naturally appear in all images (**Figure 3**). Marked bands at 1,335 cm^-1^, 1,577 cm^-1^, 1,660 cm^-1^, and 2,940 cm^-1^ are associated with DNA/RNA ring breathing mode, DNA bases, protein Amide I and protein CH_3_-stretching, respectively. No difference in cell composition was observed between the 0 h (n=9) and the 24 h (n=9) control groups, confining that the chemical variation is not influenced by the time between fixing the cells. The control groups only exhibited endmember spectra associated with nucleus and cytoplasmic content (**Figures 3A, B**). The same was the case for cells exposed to FaDOH for 1 h (n=9). However, the spectral unmixing analysis of cells treated with FaDOH for 5 h (2 out of n=9) and all cells treated with FaDOH for 24 h (n=16) resulted in additional endmember spectra. These endmember spectra represent a high degree of lipid content (red endmember) with characteristic bands at 1,263 cm^-1^, 1,745 cm^-1^, 2,850 cm^-1^, and 2,885 cm^-1^ (=CH deformation, C=O stretch, symmetric and antisymmetric CH_2_-stretch, respectively) (**Figures 3C, D**). These bands are associated with most types of lipids, although the relatively high intensity of the band around 1,665 cm^-1^ (C=C stretching) which equals the band intensity around 1,445 cm^-1^ (CH_2_ deformation) indicates a high degree of unsaturation of fatty acid residue (Czamara et al., 2015). It should be noted that the level of unsaturation varied among the individual lipid endmember spectra which in general may reflect the presence of a variety of types of lipids. Corresponding false colour images showed that the lipids (red) primarily resided in the cytoplasm (**Figure 3C**). The second green endmember in **Figure 3D** assigned to cytoplasm is relatively low in intensity and is only representative of low intensity and not necessarily spectral content. No FaDOH specific spectral features were observed in any spectra suggesting that either the concentration within the cell is below the detection limit or it is metabolized to untraceable amounts within 1 h.

**Figure 4:**
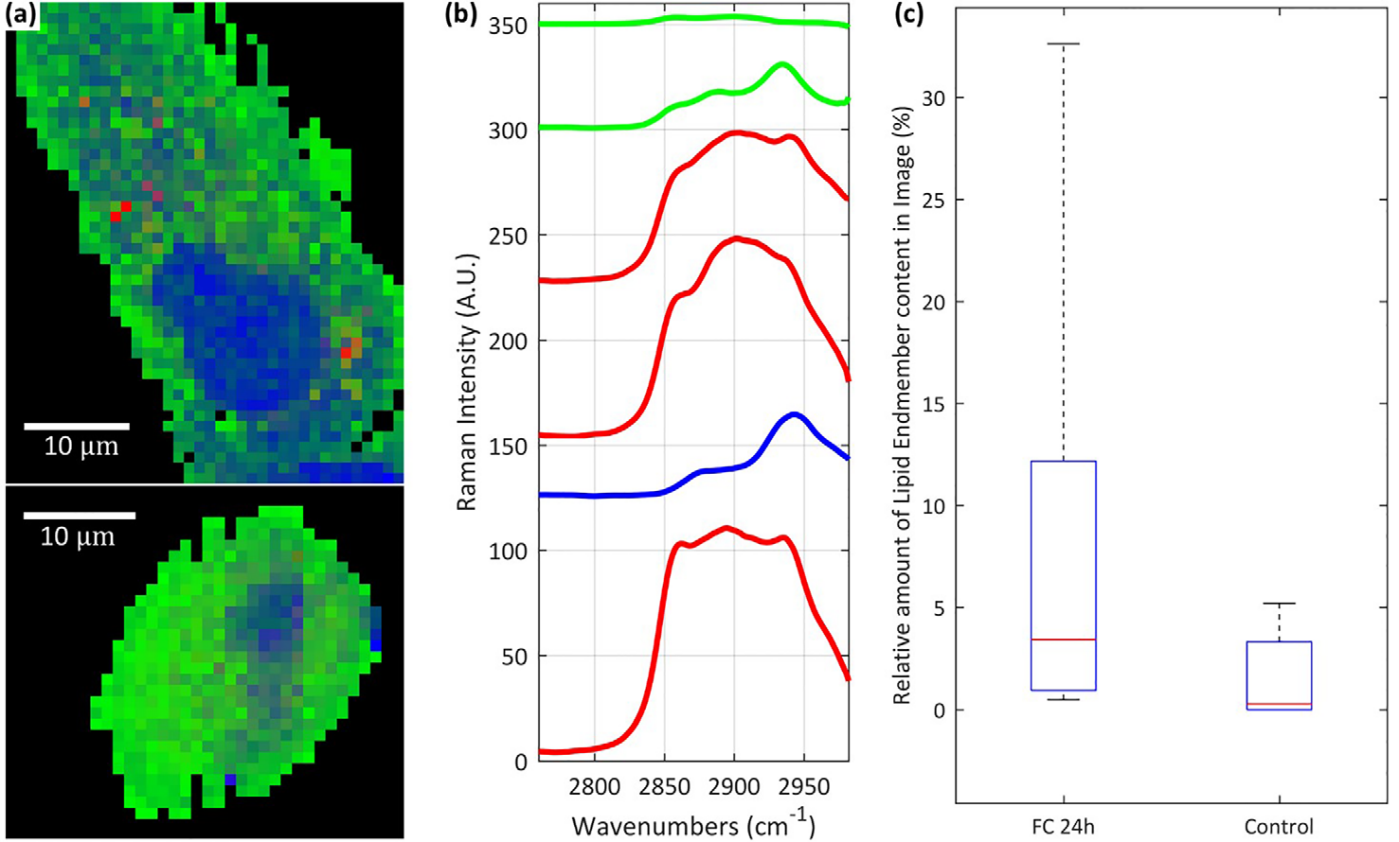
Raman analysis of lipid content in cells. Representative examples of false colour images **(A)** of falcarindiol (FaDOH) 24 h cell (upper) and control cell (lower) produced using the spectral unmixing algorithm N-FINDR on the combined set of spectral data containing all FaDOH 24 h cells and control cells. The unmixing analysis was focused on the CH-stretch spectral region along with 6 endmembers **(B)**. Box plot representing relative amount of lipid content in FaDOH 24 h (FC 24h) and control cells **(C)** calculated using relative area quantification on the false colour images and an abundance value threshold of 0.2. The lipid content in cells is identified using the red endmember spectra in (blue and green represent nucleus and cytoplasm respectively) **(B)**. Scale bars are 10 µm.

A quantitative comparison of chemical variance between cells exposed to FaDOH and control cells were conducted for the 24-h timepoint where all cells, which had been treated with FaDOH individually expressed high lipid content (**Figure 4**). Combining the spectra from images of cells treated with FaDOH for 24 h and 24-h control cells into one data matrix and applying the spectral unmixing N-FINDR analysis using six endmembers (**Figure 4B**) allowed for relative area quantification of the chemical content (**Figure 4C**). Only a reduced set of cell maps of a certain quality were considered for the analysis (FaDOH: n=8, control: n=5). The quality was evaluated by examining the influence of background, noise and whether the map contained enough of the cell area. The analysis was focused on the wavenumber region of the CH-stretch (2,760 cm^-1^ to 2,982 cm^-1^) due to the large signal to noise ratio. False colour images were reconstructed based on the colours assigned to the six endmembers common for the entire dataset (**Figure 4A**). Three of the six endmember spectra were associated with lipids (red), one was associated with components of the nucleus (blue), and two with cytoplasmic content (green) (**Figure 4B**). From each false colour image, the amount of lipid in the cells were quantified by relating the number of pixels with a red abundance value of 0.2 or higher to the total number of pixels constituting each cell, i.e., black pixels were not included. The average amount of lipid in cells treated with FaDOH for 24 h and control cells were then calculated and compared (**Figure 4C**). The results showed a trend toward more lipid content in treated cells compared to untreated cells, although not significant due to the large cell to cell variance.

**Figure 5:**
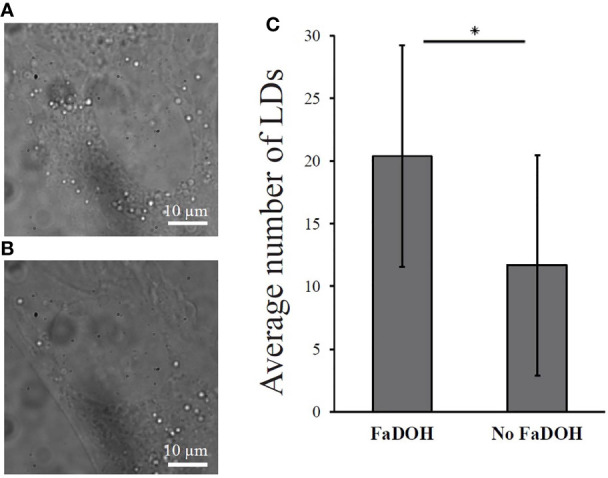
White light microscopy analysis of lipid droplets (LDs) in cells. The amount of lipid droplets increases significantly with treatment of 5 μM falcarindiol (FaDOH). **(A)** Representative white light microscopy image of human mesenchymal stem cell (hMSC) treated with 5 μM FaDOH (24 h) with which LDs can be visualized. **(B)** Representative white light microscopy images of LDs in untreated (24 h) hMSC. **(C)** LD count of the white light microscopy images. Bar graph represent mean ± SD. LDs were counted in 50 cells in different images for each set of samples. * indicates *P* < 0.05 in a non-parametric Wilcoxon Rank Sum test. Scale bars are 10 µm.

In order to confirm the increased lipid content, white light microscopy was conducted in both untreated control and FaDOH treated hMSCs. White light microscopy is a microscopy technique with low phototoxicity which can be used for detection of LDs because of the higher refractive index of LDs compared to the surrounding cytoplasm (Lyn et al., 2010). Fifty cells for each condition were imaged and the number of LDs were quantified in each cell. LDs are shown as the bright white dots surrounding the nucleus of the cell (**Figures 5A, B**). The quantification showed significant differences in the number of LDs, confirming an increased number in the FaDOH treated hMSCs (**Figure 5C**).

### FaDOH Upregulates PPARγ2 Expression in HT-29 Cells

**Figure 6:**
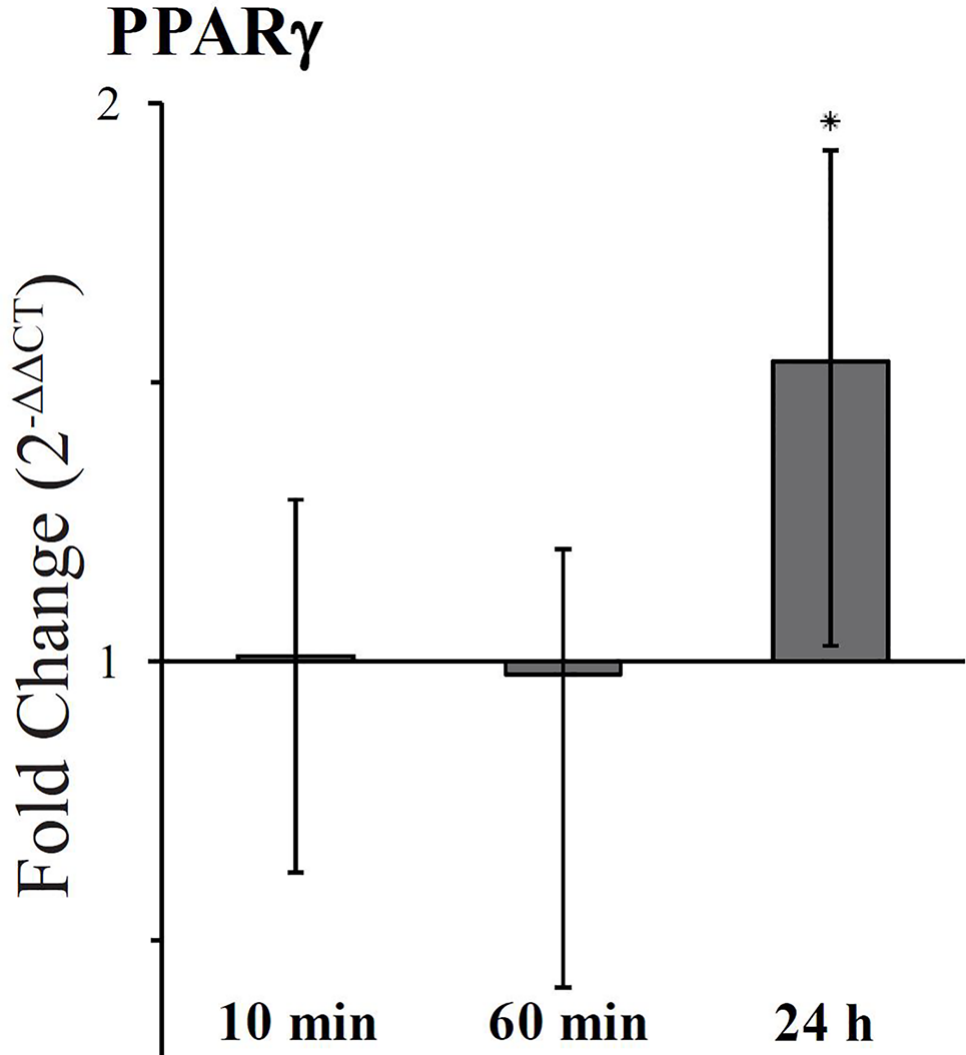
Fold change expression of PPARγ2 in cells treated with falcarindiol (FaDOH) relative to control with no treatment. Ribosomal protein large P0 (RPLP0) was used as house-keeping gene in the qPCR quantification. Bars represent mean ± SD. * indicates *P* < 0.05.

The expression level of the isoform PPARγ2, predominantly present in adipose tissue and intestine (Saraf et al., 1998), was examined in HT-29 cells treated with 5 µM FaDOH for 10 min, at 1 h and 24 h. At 10 min and 1 h treatment it was tested, if there was an immediate response, and the expression level at 24 h was studied since changes had been observed with Raman spectroscopy at this timepoint. The RT-qPCR analyses are calculated as fold change compared to cells with no treatment (**Figure 6**). No immediate response was observed for PPARγ2 (10 min and 1 h), however a significant upregulation (P<0.05) was observed after 24 h.

### Diet Supplemented With FaDOH and FaOH Increase ABCA1 Expression in Neoplastic Tissue in Rats

**Figure 7:**
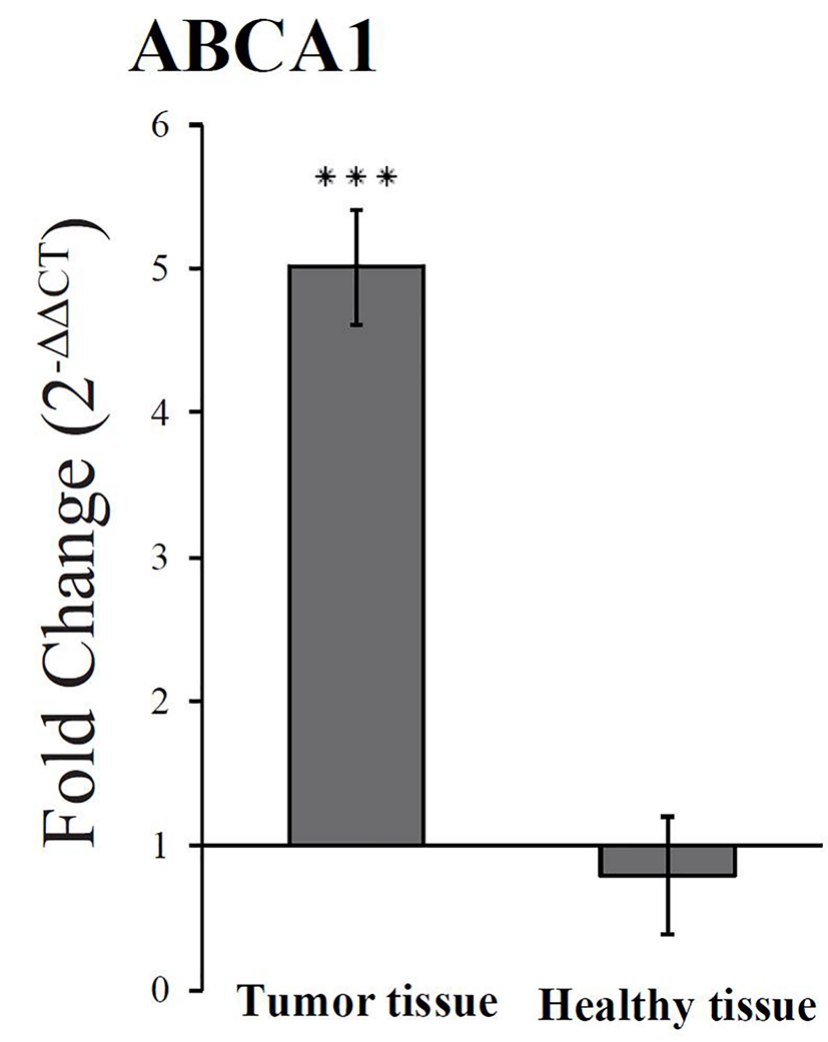
An upregulated expression of ABCA1 was observed in neoplastic colon tissue from rats receiving falcarinol (FaOH) and falcarindiol (FaDOH) in the diet compared to the neoplastic colon tissue from rats not receiving FaOH and FaDOH in the diet (control). This upregulation is neither seen in healthy colon tissue from rats receiving FaOH and FaDOH in the diet or receiving only a control rat diet. Dark gray bars represent neoplastic tissue and light grey bars represent healthy tissue. All values represent the mean ± SD. ***P < 0.001.

A previous study of the effect of dietary FaDOH and FaOH in neoplastic tissue in a rat model did show a downregulation of PPARγ as opposed to the current study in HT-29 cells. The downregulation was unexpected, but could be due to the complexity of the tissue sample, since the neoplastic cells in the tissue sample could be affected by microbiota and the cells in the surrounding tissue (Kobaek-Larsen et al., 2019). However the activation of PPARγ can lead to increased ABCA1 gene transcription in macrophages (Chawla et al., 2001) and ABCA1 has shown to have anticancer activity in colon cancer cells (Smith and Land, 2012). Therefore, the effect of FaDOH and FaOH on ABCA1 gene regulation was studied in rats receiving a standard rat diet (SRD) supplemented with 7 µg FaDOH g^-1^ feed and 7 µg FaOH g^-1^ feed compared with rats receiving SRD without supplement (**Figure 7**). RT-qPCR analyses showed no significant difference in the expression level in healthy tissue for ABCA1 when the rats received FaOH and FaDOH as a food supplement compared to rats receiving only SRD. However, a significant upregulation in the expression level was detected when comparing biopsies of neoplastic tissue from rats receiving a SRD supplemented with FaOH and FaDOH with biopsies of neoplastic tissue from rats receiving SRD.

## Discussion

The mechanism by which FaDOH promotes cancer cell death has been suggested to be due to excessive endoplasmic reticulum (ER) stress (Jin et al., 2012). The high degree of unsaturation of the fatty acids observed in some lipid endmember spectra may be due to the presence of cholesteryl esters such as cholesteryl oleate and lineolate (Czamara et al., 2015), which are major constituents of LDs. LDs are part of the lipid and cholesterol homeostasis in the cell. Cholesterol is synthesized and esterified in the ER before being transported to other sites in the cell (Wustner and Solanko, 2015). Different stress factors such as nutrient deprivation, mitochondrial dysfunction, oxidative stress, lipid overload, chemotherapy and ER stress results in LD biogenesis (Petan et al., 2018). Therefore, it is plausible that the ER stress leads to the increased formation of LDs, we observe in hMSCs treated with FaDOH. The unregulated growth by cancer cells often leads to ER stress due to insufficient amount of cholesterol and unsaturated lipids (Young et al., 2013). ER stress has, e.g., been observed for clear-cell renal carcinoma (ccRCC), which also has an increased formation of LDs (Qiu et al., 2015). The increased ER stress by FaDOH therefore makes cancer cells more vulnerable (Jin et al., 2012). Furthermore, the increased expression of ABCA1 could lead to further exhaustion of cholesterol from cancer cells. This correlates with the lower viability we observed for HT-29 cells treated with FaDOH as compared to hMSCs (**Figure 1**).

There is a correlation between the ability of the cells to form LDs and the chemoresistance of the cells, and HT-29 is a cell line with a high content of LDs (Cotte et al., 2018). The formation of LDs protects the cell against lipid induced ER stress (Bosma et al., 2014). The nuclear receptor PPARγ is associated with genes involved in lipid metabolism and in LDs formation (Poulsen et al., 2012) and as FaDOH is a partial PPARγ agonist (El-Houri et al., 2015a) the increased LD formation seen in the hMSCs could be linked to the partial PPARγ agonist activity of FaDOH.

The expression of PPARγ is downregulated in cancer cells (Lecarpentier et al., 2017) and PPARγ expression has been found to be an independent prognostic factor for overall and gastric cancer specific mortality in patients with intestinal-type gastric cancer with PPARγ-positive tumors resulting in lower overall and cancer-specific mortalities than PPARγ-negative tumors (Cho et al., 2015). It is already known that PPARγ can suppress tumorigenesis through regulation and interaction with β-catenin (Girnun et al., 2002; Jansson et al., 2005), and that PPARγ agonist inhibits the development of colon cancer (Brockman et al., 1998; Sarraf et al., 1998). The present study has revealed that FaDOH is able to upregulate PPARγ expression in HT-29 cells (**Figure 6**). Thus, FaDOH could have multiple effects on cancer cells from upregulation of lipid uptake, to increased ER stress, both which could lead to the increased LD formation we observe, but FaDOH could also have anticancer effect by downregulation of β-catenin through PPARγ.

It has previously been shown that FaDOH increases cholesterol efflux, partly *via* expression of ABCA1 (Wang et al., 2017) that is induced by PPARγ (Chawla et al., 2001; Mogilenko et al., 2010; Wang et al., 2017). ABCA1 is important for the formation of HDL and thereby cholesterol removal (Wang and Tall Alan, 2003). Since increased synthesis of cholesterol (Pihlajamäki et al., 2004) and downregulation of ABCA1 (Patel et al., 2011) is linked to type 2 diabetes FaDOH could have antidiabetic properties as a PPARγ agonist both by stimulating glucose uptake (El-Houri et al., 2015a) and increasing cholesterol removal through upregulation of ABCA1. Cholesteryl esters is found naturally in LDs (Fujimoto and Parton, 2011) and the fact that endmember spectra of Raman spectroscopy indicates the presence of cholesteryl oleate and lineolate upon FaDOH treatment, could have important implication for the function of FaDOH, since linoleic acid has been linked to antidiabetic function (Wu et al., 2017). Further studies are required to investigate and prove this connection.

In conclusion, FaDOH induces change in lipid content in hMSCs exposed to sub-toxic amounts of FaDOH as observed using label-free Raman spectroscopic mapping and white light microscopy. Cells treated with FaDOH show a significant upregulation of LDs compared to control cells, and Raman spectroscopy indicated the formation of cholesteryl lineolate. RT-qPCR showed increased expression of PPARγ2 in cancer cells and increased expression of ABCA1 in neoplastic tissue, which could indicate an increased formation of LDs in cancer cells and normal cells, thus contributing to the understanding of the anticancer and antidiabetic properties of FaDOH. The involvement of PPARγ in the upregulation of ABCA1 by FaDOH and the formation of LDs cannot be excluded based on our results and therefore further investigations in cells of normal and cancer origin are needed, which may include treatment with PPARγ antagonists to explore the potential anticancer and antidiabetic properties of FaDOH.

## Data Availability Statement

The raw data supporting the conclusions of this article will be made available by the authors, without undue reservation, to any qualified researcher.

## Ethics Statement

The animal study was reviewed and approved by Central Animal Experimentation Inspectorate in Denmark.

## Author Contributions

CA, LC, AR, EP-O, MA, MH, and EA wrote the main manuscript text, and CA, AR, and EP-O prepared **Figures 1**−**7**. CA, AR, EP-O, MN, SJ, ME, AL, RC, JL, DH, JJ, MA, and EA performed the experiments and data analysis. MH provided detailed advice on Raman spectroscopy and performed image data analysis. RE-H provided purified falcarindiol. MK-L performed animal experiments and autopsy. All authors contributed to the article and approved the submitted version.

## Funding

We thank the Carlsberg foundation - CF14-0786 (to EA), the Villum Kann Rasmussen foundation - grant number 19105 (to EA) and the Independent Research Fund Denmark Project - 7017-00163 (to MH and MA) for financial support.

## Conflict of Interest

The authors declare that the research was conducted in the absence of any commercial or financial relationships that could be construed as a potential conflict of interest.
